# Functions of N6-methyladenosine and its role in cancer

**DOI:** 10.1186/s12943-019-1109-9

**Published:** 2019-12-04

**Authors:** Liuer He, Huiyu Li, Anqi Wu, Yulong Peng, Guang Shu, Gang Yin

**Affiliations:** 1 Department of Pathology, Xiangya Hospital, School of Basic Medical Sciences, Central South University, Changsha, 410008 Hunan Province China; 2 School of Basic Medical Sciences, Central South University, Changsha, 410013 Hunan Province China

**Keywords:** N6-methyladenosine, writer, eraser, reader, cancer

## Abstract

N6-methyladenosine (m6A) is methylation that occurs in the N6-position of adenosine, which is the most prevalent internal modification on eukaryotic mRNA. Accumulating evidence suggests that m6A modulates gene expression, thereby regulating cellular processes ranging from cell self-renewal, differentiation, invasion and apoptosis. M6A is installed by m6A methyltransferases, removed by m6A demethylases and recognized by reader proteins, which regulate of RNA metabolism including translation, splicing, export, degradation and microRNA processing. Alteration of m6A levels participates in cancer pathogenesis and development via regulating expression of tumor-related genes like BRD4, MYC, SOCS2 and EGFR. In this review, we elaborate on recent advances in research of m6A enzymes. We also highlight the underlying mechanism of m6A in cancer pathogenesis and progression. Finally, we review corresponding potential targets in cancer therapy.

## Background

Post-transcriptional modification has emerged as an important regulator of a variety of physiological processes and disease progression, attracting accumulating attention in bioscience research. Among numerous RNA modifications, N6-methyladenosine (m6A) is the most abundant mRNA modification. An average of 1000 nucleotides are found to contain 1–2 m6A residues [1, 2]. Mainly occurring in the RRACH sequence (where R = A or G, H = A, C, or U) [3, 4], m6A is enriched near the stop codon, 3′ untranslated region (UTR) and long internal exon [5, 6]. M6A can also be found in RNA of bacteria and viruses [7, 8].

M6A can be installed by the methyltransferase complex (MTC) and removed by demethylases [9, 10]. M6A alters target gene expression, thus influencing the corresponding cell processes and physiological function. In molecular mechanism, m6A participates in almost all steps of RNA metabolism including mRNA translation, degradation, splicing, export and folding [11, 12]. Roles of m6A in various cancers have been reported recently [87,88,]. In this review, we focus on the up-to-date progress in m6A enzymes. We describe functions of m6A in tumorigenesis and cancer progression. Finally, we discuss future research directions of m6A.

## Regulators of m6A

**Fig. 1:**
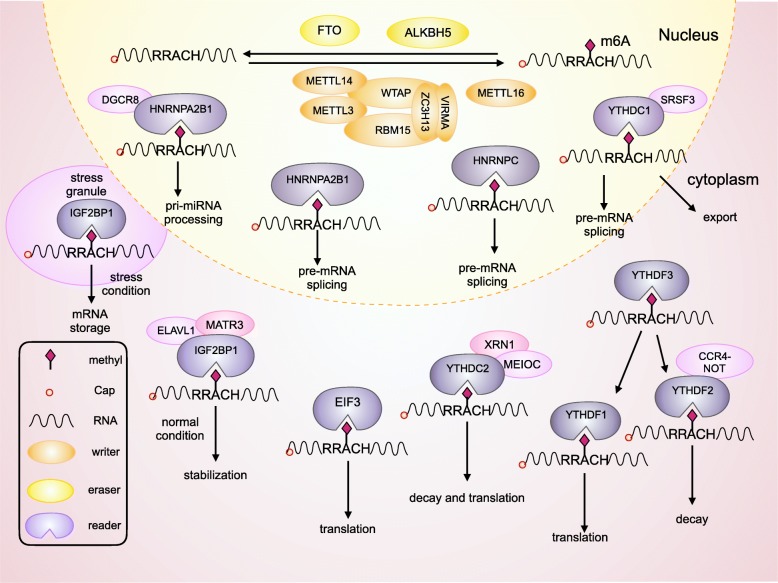
Mechanism of m6A. The m6A methylation is catalyzed by the writer complex including METTL3, METTL14, WTAP, VIRMA, RBM15, and ZC3H13. The m6A modification is removed by demethylase FTO or ALKBH5. Reader proteins recognize m6A and determine target RNA fate.

Regulators of m6A can be divided into 3 types: writers, erasers and readers. M6A is catalyzed by the methyltransferase complex (MTC), also called “writers”. Demethylase, also termed as “eraser”, removes m6A. RNA reader protein recognizes m6A, binds the RNA and implements corresponding functions (Table 1, Fig. 1). Crosslink among writers, erasers and readers, is involved in cancer pathogenesis and progression [35, 36].

**Table 1:** Functions of m6A regulators in RNA metabolism.

Type	Regulator	Function	Reference
m6A writer	METTL3	catalyzes m6A modification	[13, 14]
	METTL14	helps METTL3 to recognize the subtract	[13, 14]
	METTL16	catalyzes m6A modification	[15]
	RBM15	binds the m6A complex and recruit it to special RNA site	[16, 17]
	VIRMA	recruits the m6A complex to the special RNA site and interacts with polyadenylation cleavage factors CPSF5 and CPSF6	[18]
	WTAP	contributes to the localization of METTL3-METTL14 heterodimer to the nuclear speckle	[19]
	ZC3H13	bridges WTAP to the mRNA-binding factor Nito	[20]
m6A eraser	ALKBH5	removes m6A modification	[21]
	FTO	removes m6A modification	[22]
m6A reader	EIF3	enhances mRNA translation	[23]
	HNRNPA2B1	mediates mRNA splicing and primary microRNA processing	[24]
	HNRNPC	mediates mRNA splicing	[25]
	IGF2BPs	enhances mRNA stability and storage	[26]
	YTHDC1	contributes to RNA splicing and export	[27, 28]
	YTHDC2	enhances the translation of target RNA and reduces the abundance of target RNA	[29]
	YTHDF1	enhances mRNA translation	[30]
	YTHDF2	promotes mRNA degradation	[31, 32]
	YTHDF3	enhances translation and degradation by interacting with YTHDF1 and YTHDF2	[33, 34]

### Writers

M6A is installed co-transcriptionally through the methyltransferase complex (MTC) that consists of METTL3 catalytic subunit and other accessory subunits including METTL14, WTAP, VIRMA, RBM15, and ZC3H13 [37]. METTL14 forms a stable complex with METTL3 and plays a key role in substrate recognition [13, 14, 38]. Wilms Tumor 1 associated protein (WTAP) ensures the localization of the METTL3-METTL14 heterodimer to the nuclear speckle and promotes catalytic activity [16, 19]. RNA binding motif 15 (RBM15) binds the m6A complex and recruits it to special RNA site [17, 39]. ZC3H13 enhances m6A through bridging WTAP to the mRNA-binding factor Nito [20, 40]. VIRMA directs m6A in 3′ UTR and near stop codon by recruiting the MTC to modulate region-selective methylation [18]. In addition, METTL16 is a newly discovered writer that catalyzes m6A modification in U6-snRNA and participates in pre-RNA splicing [15].

#### METTL3

In glioblastoma (GBM), METTL3 exerts an oncogenic effect through modulating nonsense-mediated mRNA decay of splicing factors and alternative splicing isoform switches. Loss of METTL3 results in higher level of BCL-XS isoform and NCOR2α isoform and inhibition of GSC growth and self-renewal [41]. In gastric cancer, up-regulated METTL3 promotes stability of ZMYM1, thus enhancing EMT process in vitro and metastasis in vivo [42]. Moreover, METTL3 can also participate in regulation of target mRNA in a post-modification way, therefore partially acting as a reader [43, 44].

METTL3 modulates hematopoietic stem cells (HSC) self-renewal through enhancing expressions of self-renewal-related genes such as Nr4a2, p21, Bmi-1 and Prdm16 [45]. Depletion of METTL3 leads to a significant suppression of HSC reconstitution potential [46].

In human non-small cell lung carcinoma, METTL3, which undergoes sumoylation, has a reduced ability to catalyze m6A, resulting in enhanced tumorigenesis [47–49].

R-loops are three-stranded nucleic acid structures. M6A enhances co-transcriptional R-loops, impairing readthrough activity of Pol II for efficient termination [50].

METTL3 participates in neurogenesis and development through installing m6A in histone methyltransferase Ezh2 and enhancing expression of it [51].

METTL3 suppresses autophagic flux and enhances apoptosis in hypoxia/reoxygenation -treated cardiomyocytes by installing m6A in TFEB and inhibiting its expression, an essential regulator of lysosomal biogenesis and autophagy genes [52].

METTL3 facilitates the stability of viral RNA-dependent RNA polymerase 3D and boosts type 71 enterovirus replication by inducing sumoylation and ubiquitination of the polymerase [53].

#### METTL14

METTL14 is markedly elevated in Epstein–Barr virus (EBV) latently infected cells, promoting cell proliferation. Mechanistically, METTL14 contributes to oncogenesis through inducing m6A on the essential EBV latent antigen EBNA3C and thereby enhancing expression and stability of it. Interestingly, EBNA3C also increases expression and stability of METTL14 [54].

METTL14 participates in erythropoiesis by promoting the translation of related genes including those coding for SETD histone methyltransferases, ribosomal components, and polyA RNA binding proteins [55].

Histone H3 trimethylation at H3K36me3 (Lys36) mediates m6A deposition, which can be recognized and bound by METTL14. METTL14 promotes the binding of the MTC to adjacent RNA polymerase II, therefore transferring the MTC to actively transcribed nascent RNAs [56].

METTL14 mediates the self-renewal capabilities of HSCs through promoting the expression of self-renewal-related genes such as Bmi-1 and Prdm16 [45].

METTL14 regulates post-implantation embryonic development via promoting the conversion from naive state to primed state of the epiblast. Loss of METTL14 impairs the priming and further differentiation competence of embryonic stem cells [57]. Moreover, the role of METTL14 in regulation of cancer stem cell has been revealed [58].

#### METTL16

METTL16 binds a subset of mRNAs and methylates long noncoding RNA (lncRNA) and U6 small nuclear RNA (U6 snRNA) [59, 60]. The UACAGAGAA sequence is required for METTL16-mediated-methylation and the N-terminal module of METTL16 is essential for RNA binding [61, 62].

METTL16 regulates S-adenosylmethionine (SAM) homeostasis. SAM is an important methyl-group donor in DNA and histone methylation. MAT2A encodes the SAM synthetase. Under loss-of-SAM conditions, METTL16 induces the splicing of a retained intron, thus promoting expression of MAT2A and level of SAM [63].

METTL16 participates in catalyzing m6A in A43 of the U6 small nuclear RNA [15].

### Erasers

Employing ferrous iron as cofactor and α-ketoglutarate as cosubstrate, eraser removes m6A, thereby functioning as demethylase [64].

#### FTO

FTO not only controls mRNA splicing by inhibiting SRSF2 binding at splice sites [65], but also regulates adipogenesis by increasing the relative expression of the pro-adipogenic RUNX1T1-S isoform [66]. FTO blocks YTHDF2-mediated mRNA degradation by reducing m6A levels of cyclin A2 and cyclin-dependent kinase 2, thereby promoting fat cell cycle progression and adipogenesis [67].

FTO regulates leukemogenesis by modulating proliferation, differentiation and apoptosis of acute myeloid leukemia (AML) cells [68].

In melanoma, FTO impairs IFNγ-induced killing in melanoma cells in vitro via up-regulating PD-1, CXCR4, and SOX10 through suppressing YTHDF2-mediated-degradation and inhibits response to anti-PD-1 blockade immunotherapy [69].

#### ALKBH5

In head and neck squamous cell carcinomas, ALKBH5 is up-regulated and promotes cell viability [70].

In epithelial ovarian cancer (EOC), up-regulated ALKBH5 impairs the autophagy and facilitates cell proliferation and invasion [71].

ALKBH5-directed m6A demethylation is involved in splicing and the production of longer 3′ -UTR mRNAs [72, 73].

### Readers

**Fig. 2:**
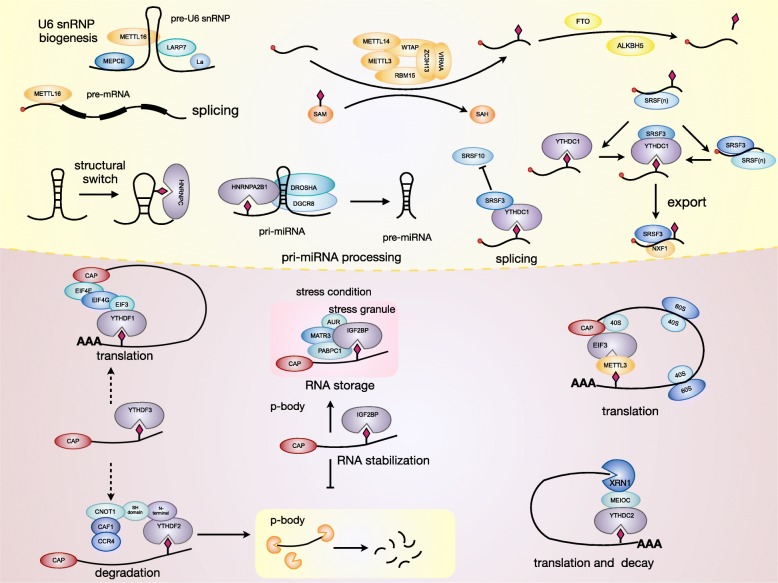
Functions of readers on RNA. Readers participate in a variety of steps in RNA metabolism including translation, splicing, export, degradation and so on.

“Readers” recognize and bind m6A sites, leading to different destinies of target RNA [74, 75] (Fig. 2). In YTH domain family, the YT521-B homology (YTH) domain functions as the module for recognizing m6A [5, 76, 77]. YTHDF1 enhances mRNA translation and protein synthesis through interacting with initiation factors [30]. YTHDF2 induces degradation of the transcripts by selectively binding m6A-modified mRNA and recruiting them to mRNA decay sites [32]. YTHDF3 respectively enhances RNA translation via interacting with YTHDF1 and promotes RNA degradation by associating with YTHDF2 [33, 34]. YTHDC1 contributes to RNA splicing and export [27, 28]. YTHDC2 increases the translation efficiency of target RNA but reduces the abundance of them [29]. Recently, insulin-like growth factor 2 mRNA-binding proteins (IGF2BPs) including IGF2BP1–3, are identified to bind m6A and function as readers. IGF2BPs promote RNA expression by enhancing RNA stability [78–80]. Eukaryotic initiation factor 3 (EIF3) facilitates cap-independent translation [23]. HNRNP (heterogeneous nuclear ribo nucleo protein) A2B1 mediates the alternative splicing of target RNAs and enhances primary miRNA processing by interacting with the microRNA microprocessor complex protein DGCR8 [24], while HNRNPC participates in pre-mRNA processing [25, 81–84].

#### YTHDF1

Tumor neoantigens are involved in anti-tumor immune response and immunotherapy. YTHDF1 promotes lysosomal proteases-directed-degradation of neoantigens in dendritic cells by recognizing their m6A modification and enhancing their translation, thereby suppressing dendritic cells presenting tumor neoantigens to T cells and promoting tumor cells to evade immunosurveillance [85].

#### YTHDF2

YTHDF2 recruits the CCR4–NOT deadenylase complex by a direct association between the N-terminal region of YTHDF2 and the SH domain of the CNOT1 subunit, initiating deadenylation and decay of m6A-containing mRNAs [31]. Moreover, YTHDF2 mediates m6A methylated RNA endoribonucleolytic cleavage through forming a complex with HRSP12 and RNase P/MRP [86].

YTHDF2 interacts with miRNA [87]. In prostate cancer, YTHDF2 expression can be suppressed by miR-493-3p, therefore impairing cell proliferation and migration [88].

YTHDF2 affects the homeostasis of HSC. YTHDF2 deficiency prevents degradation of mRNAs of both survival-related genes and Wnt target genes during hematopoietic stresses, which synergistically enhances the regenerative capacity of HSCs [89].

M6A inhibits circular RNA (circRNA) immunity through functioning as a marker for “self”. Unmodified circRNA forms a complex with activated pattern recognization receptor RIG-I and K63-polyubiquitin, resulting in MAVS filamentation, IRF3 dimerization, and interferon production. YTHDF2 suppresses circRNA immunity by binding m6A-modificated circRNA, leading to the “self circRNA” being ignored and failure of RIG-I activation [90].

YTHDF2 recognizes and binds the nuclear receptor peroxisome proliferator-activator a (PPaRa), leading to decay of PPaRa [91].

#### YTHDF3

LncRNA GAS5 enhances YAP phosphorylation to increase ubiquitination and degradation of it, thereby impairing YAP signaling and inhibiting colorectal cancer (CRC) cell proliferation and metastasis. YTHDF3 recognizes m6A modified GAS5 and induces decay of it [92].

CircRNAs regulate numerous biological processes [93]. M6A participates in efficient initiation of protein translation from circRNAs in cells, requiring initiation factor eIF4G2 and YTHDF3 [94].

In seminoma, YTHDF3 and VIRMA are significantly overexpressed and there is a positive correlation between their expression [95].

#### YTHDC1

M6A level is higher in RNA X-inactive specific transcript (XIST). YTHDC1 recognizes m6A in XIST and regulates XIST function [17].

M6A of MAT2A mediated by METTL16 is read by YTHDC1. Inhibition of YTHDC1 and METTL16 damages SAM-responsive modulation of MAT2A [96].

#### YTHDC2

YTHDC2 is an RNA-induced ATPase with a 3′ -to-5′ RNA helicase activity [97]. Germ cells down-regulated YTHDC2 enter meiosis but process prematurely to abnormal metaphase and apoptosis [98, 99].

#### IGF2BPs

IGF2BPs enhance expression of target mRNA by promoting the stability of target mRNA through binding to mRNA stabilizers like ELAVL1 and MATR3 under normal conditions and by promoting the storage of target mRNA through translocating to stress granules under stress condition [26].

IGF2BP1 promotes tumor cell malignant phenotype by preferentially associating upstream of miRNA binding sites in the 3′ UTR of target mRNAs and then antagonizing miRNA-impaired gene expression [100]. Serum response factor (SRF) controls expression of oncogenes like FOXK1 and PDLIM7. IGF2BP1 can inhibit the microRNA-mediated degradation of SRF expression and thereby acting as a cancer promoter [78, 101]. In metastatic melanomas, expression of sequestosome 1/SQSTM1/p62 (p62) is higher, associated with poor patient prognosis. Mechanistically, p62 enhances stability of FERMT2 and other pro-metastatic factors through interacting with IGF2BP1 [102]. In HCC, TCAM1P-004 interacts with IGF2BP1, thereby suppressing IGF2 translation and enhancing DDIT3 expression, resulting in inhibition of cell proliferation and promotion of cell apoptosis [103].

In colorectal carcinoma, IGF2BP2 recognizes m6A in the coding sequence (CDS) regions of target gene SOX2 and prevents it degradation, contributing to colorectal cancer pathogenesis and progression [80].

IGF2BP3 shows oncogenic features and is markedly up-regulated in a variety of cancer types, associated with poor patient survival. IGF2BP3 functions as a potential oncogene across multiple cancer types [104]. In pancreatic cancer, the DNA methylation level of IGF2BP3 is significantly reduced and the expression IGF2BP3 is higher, associated with patient overall survival [105]. IGF2BP3 participates in the fetal–adult hematopoietic switch through interacting with RNA-binding protein Lin28b. In B-cell progenitors, Lin28b and IGF2BP3 promote stability of mRNAs such as B-cell regulators Pax5 and Arid3a [79].

#### EIF3

EIF3 is essential for specialized translation initiation through interacting with the 5′ cap, contributing to assembly of translation initiation complexes on eIF3-specialized mRNA [106].

EIF3 is highly expressed in colorectal cancer and contributes to mTOR signaling. EIF3 knockdown promotes autophagy [107].

In head and neck squamous cell carcinoma, DDX3 promotes the association of the cap-binding complex with mRNAs and enhances recruitment of EIF3, thereby contributing to the expression of metastatic-related gene expression [108].

## Roles of m6A in cancer

**Fig. 3:**
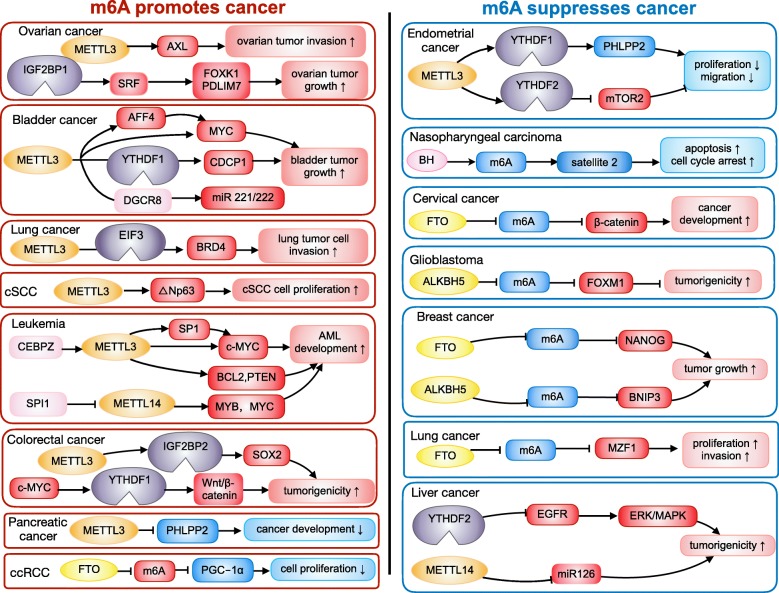
The role of m6A in cancers. The effect of m6A on cancer is reflected in the regulation of cancer-related gene expression. The m6A modification promotes cancer pathogenesis and progression through enhancing oncogene expression and inhibiting tumor suppressor gene expression. The m6A modification hampers cancer pathogenesis and progression through inhibiting oncogene expression and enhancing tumor suppressor gene expression.

Effect of m6A on cancer is reflected in the change of these tumor-related genes. Systematic study of the crosslink between substrate genes, m6A modification and post-modification regulation will contribute to reveal the mechanism of m6A in cancer comprehensively (Table 3) [116, 117]. Recently, many studies choose one or two of m6A regulators to explore its aberrant expression and underlying mechanism in cancer (Table 2). Here, we detail the role of m6A in cancer pathogenesis and progression (Fig. 3).

**Table 2:** Roles of m6A enzymes in cancer

cancer type	enzyme	target RNA	effect of enzyme on target RNA	role of m6A in cancer	reference
AML	METTL3	c-MYC, BCL2, PTEN	translation	inhibits cell differentiation and apoptosis, promotes leukemia progression	[118]
	METTL3	SP1	translation	promotes cell proliferation and inhibits cell differentiation	[119]
Breast cancer	ALKBH5	NANOG	stabilization	inhibits tumor formation and breast cancer stem cell population	[120]
	FTO	BNIP3	degradation	inhibits breast cancer cell proliferation, colony formation and metastasis	[121]
	METTL3	HBXIP	expression	enhances proliferation, invasion and metastisis and inhibits apoptosis	[122]
CSCC	FTO	β-catenin	expression	enhances the chemo-radiotherapy resistance	[123]
GSC	ALKBH5	FOXM1	expression	impairs proliferation and tumorigenicity	[21]
	METTL3	SOX2	stability	enhances of neurosphere formation	[124]
HCC	METTL14	miRNA 126	splicing	inhibits the migration and invasiveness of HepG2 cells and restrains tumor metastasis	[125]
LUSC	FTO	MZF1	stability	inhibits proliferation and invasion and increases apoptosis	[22]
Ovarian cancer	METTL3	AXL	translation	promotes growth and invasion of ovarian tumors	[126]
Pancreatic cancer	ALKBH5	KCNK15-AS1	expression	promotes migration and invasion	[127]
Renal cell carcinoma	FTO	PGC-1α	stability	enhances cell growth and facilitates apoptosis	[128]

LUSC: lung squamous cell carcinoma; AML: acute myeloid leukemia; GSC: glioblastoma stem-like cell; HCC: human hepatocellular carcinoma; CSCC: cervical squamous cell carcinoma

### m6A functions as a tumor promoter

#### m6A promotes expression of oncogenes

In bladder cancer, METTL3 and oncogene CDCP1 are up-regulated, correlating with bladder cancer progression status. METTL3 elevated m6A level of CDCP1, thus promoting its translation modulated by YTHDF1 [109]. AF4/FMR2 family member 4 (AFF4) is a direct upstream regulator of MYC and can enhance MYC expression. METTL3 promotes expressions of MYC and AFF4 [129]. Inhibition of METTL3 significantly hampered bladder tumor cell proliferation, migration, invasion, and survival in vitro and impairs cell proliferation in vivo [109, 129]. METTL3 promotes maturation of pri-miR221/222 [130].

In lung adenocarcinoma, the expression of METTL3 is elevated. METTL3 enhances translation of oncogene BRD4 through forming a mRNA loop with EIF3, thus facilitating translation of the oncogene by promoting ribosome recycling, indicating that METTL3 can not only catalyze m6A but also participate in the post-methylation regulation of target mRNA therefore functioning as a reader [43]. METTL3 can also promote translation of oncogenes such as EGFR and TAZ by associating with EIF3 [44]. METTL3 depletion leads to increased cell apoptosis and decreased cell growth and invasion, impairing tumorigenicity in mouse xenografts [43, 44].

In AML, METTL3 is elevated, facilitating translation of oncogenes like SP1 through relieving ribosome stalling. SP1 regulates expression of oncogene c-MYC [119]. METTL3 enhances translation of oncogenes such as c-MYC, BCL2 and PTEN by installing m6A [118]. METTL14 is highly expressed in AML, which promotes AML development and maintenance and self-renewal of leukemia stem/initiation cells via enhancing translation and inhibiting decay of MYB and MYC [58].

In ovarian cancer, IGF2BP1 is elevated and associated with a poor prognosis. SRF contributes to tumor cell proliferation and metastasis, and transcription of oncogenes like FOXK1 and PDLIM7 [131, 132]. IGF2BP1 enhances expression of SRF through inhibiting the miRNA-mediated degradation of it in an m6A-dependent way, resulting in increased expression of SRF, FOXK1 and PDLIM7 [78, 100]. METTL3 stimulates AXL mRNA translation and epithelial-mesenchymal transition, thereby promoting growth and invasion of ovarian tumors [126, 133].

In liver cancer, levels of METTL3 and YTHDF1 are higher, related with worse overall survival. M6A in CDS of Snail enhances Snail expression through YTHDF1-mediated translation, a key transcription factor of EMT. Low level of m6A due to loss of METTL3 hampers EMT of cancer cells [115, 134].

In breast cancer, METTL3 is elevated. HBXIP (hepatitis B X-interacting protein) enhances the malignant phenotypes of breast tumor. METTL3 promotes expression of HBXIP. Interestingly, HBXIP also facilitates METTL3 expression by inhibiting miRNA let-7 g, which reduces METTL3 expression through targeting its 3′ UTR [122].

In pancreatic tumors and in smokers, expression of miR-25-3p is elevated, associated with worse prognosis. Cigarette smoke condensate promotes m6A, thus contributing to maturation of oncogene miR-25-3p in pancreatic tumor. MiR-25-3p inhibits PH domain leucine-rich repeat protein phosphatase 2 (PHLPP2), leading to the activation of oncogenic AKT-p70S6K signaling [135].

In CRC, YTHDF1 is highly expressed. Knockdown of YTHDF1 suppresses the CRC cell tumorigenicity and colonosphere formation ability through impairing Wnt/β-catenin pathway activity [136]. Moreover, c-MYC enhances YTHDF1 expression [137]. METTL3 promotes expression of SRY (sex determining region Y)-box 2 (SOX2) through IGF2BP2-directed suppression of RNA degradation [80]. In CRC cells, overexpression of RP11 due to m6A-directed-nuclear accumulation promotes cell dissemination and induces EMT [138].

In nasopharyngeal carcinoma (NPC), the m6A level of oncogenic lncRNA FAM225A is elevated, resulting in enhanced stability and higher expression of FAM225A. FAM225A exerts a cancer-promoting role through functioning as a ceRNA and sponging miR-590-3p and miR-1275, causing higher expression of ITGB3 and activation of the FAK/PI3K/Akt pathway [139]. M6A regulates NPC cell proliferation [140, 141].

In cutaneous squamous cell carcinoma (cSCC), up-regulated METTL3 promotes **△**Np63 expression, thereby enhancing cSCC cell proliferation and tumor growth [142].

#### m6A inhibits expression of tumor suppressor genes

In human hepatocellular carcinoma (HCC), highly expressed METTL3 restrains expression of SOCS2 via YTHDF2-mediated degradation [143, 144]. Depletion of METTL3 results in a decrease in cell proliferation, colony formation, and migration in vitro and inhibits tumor cell proliferation and metastasis in vivo [113]. The expression of VIRMA is also higher in HCC tissue, leading to increased cell proliferation and invasion by enhancing m6A modification and inhibiting expression of ID2 mRNA [145]. WTAP is also elevated, resulting in increased cell proliferation and colony formation through facilitating m6A in ETS1 and causing epigenetic silencing of ETS1 [146].

In clear cell renal cell carcinoma (ccRCC), higher m6A level due to less FTO inhibits expression of PGC-1α through reducing its stability. Higher level of m6A and lower expression of PGC-1α results in enhanced tumor growth [128].

In pancreatic cancer, the expression of ALKBH5 is reduced, resulting in elevated m6A level and reduced expression of tumor suppressor gene KCNK15-AS1, leading to enhanced migration and invasion of pancreatic tumor cells [127].

### m6A functions as a tumor suppressor

#### m6A inhibits expression of oncogenes

In endometrial tumors, the m6A level is lower which is caused by reduced METTL3 expression or METTL14 mutation. M6A methylation inhibits activation of the AKT pathway through YTHDF2-mediated degradation of the positive AKT regulator mTORC2. Attenuated m6A methylation leads to enhanced cell proliferation, colony formation, migration and invasion [110].

In HCC, m6A acts as a tumor suppressor through YTHDF2-directed degradation of EGFR by binding the m6A site in the 3′ UTR of this mRNA. YTHDF2 inhibits ERK/MAPK signaling cascades by destabilizing the EGFR mRNA [112].

In breast cancer, hypoxia facilitates expression of ALKBH5, resulting in reduction of NANOG mRNA m6A level and enhancement of mRNA stabilization. Elevated NANOG promotes breast cancer stem cells (BCSCs) enrichment [120].

In lung squamous cell carcinoma (LUSC), the expression of FTO is elevated. FTO facilitates oncogene MZF1 expression through inhibiting m6A methylation and increasing mRNA stabilization [22, 147].

In glioblastoma, elevated ALKBH5 reduces m6A methylation and facilitates expression of oncogene FOXM1, leading to enhancement of glioblastoma stem-like cells (GSCs) self-renewal and tumorigenicity. Furthermore, lncRNA plays a dynamic role in transcriptional and translational regulation of cancer [148]. Nuclear lncRNA FOXM1-AS promotes the association between ALKBH5 with FOXM1 nascent RNA, leading to enhanced FOXM1 expression and GSC pathogenesis [21].

#### m6A promotes expression of tumor suppressor genes

In endometrial tumors, m6A methylation inhibits activation of the AKT pathway through YTHDF1-mediated translation of the negative AKT regulator PHLPP2. Attenuated m6A methylation leads to enhanced cell proliferation, colony formation, migration and invasion [110].

In breast cancer, the expression of FTO is higher. FTO promoted breast cancer cell proliferation, colony formation and metastasis by reducing BNIP3 methylation and promoting BNIP3 degradation [121]. Premature polyadenylation (pPA) of tumor suppressor genes frequently truncates these genes, leading to inhibition of their functions. These genes such as MAGI3 undergo pPA at the intron downstream of its long internal exon, which is the m6A site. In breast cancer cells with enhanced MAGI3 pPA, m6A modification in this site is obviously diminished [149].

In HCC, Jin-zhao Ma and colleagues have found that METTL14 positively manipulates primary microRNA126 processing via an m6A-dependent manner, which impairs the metastatic potential of HCC. Overexpression of METTL14 inhibits the migration and invasiveness of HepG2 cells in vitro and restrains cell proliferation and metastasis in vivo. METTL14 not only enhances the recognition and binding of microprocessor protein DGCR8 to pri-mi126, but also promotes the subsequent processing to mature miRNA [125].

### The dual role of m6A in cancer

Accumulating evidence shows that m6A plays a dual role in cancer [150, 151]. On the one hand, m6A regulates expression of oncogenes or tumor suppressor genes, thus affecting cancer progression. On the other hand, the m6A level and m6A enzymes expression and activity can be modulated, thereby influencingz the role of m6A in cancer.

#### m6A affects cancer progression by modulating target gene

**Fig. 4:**
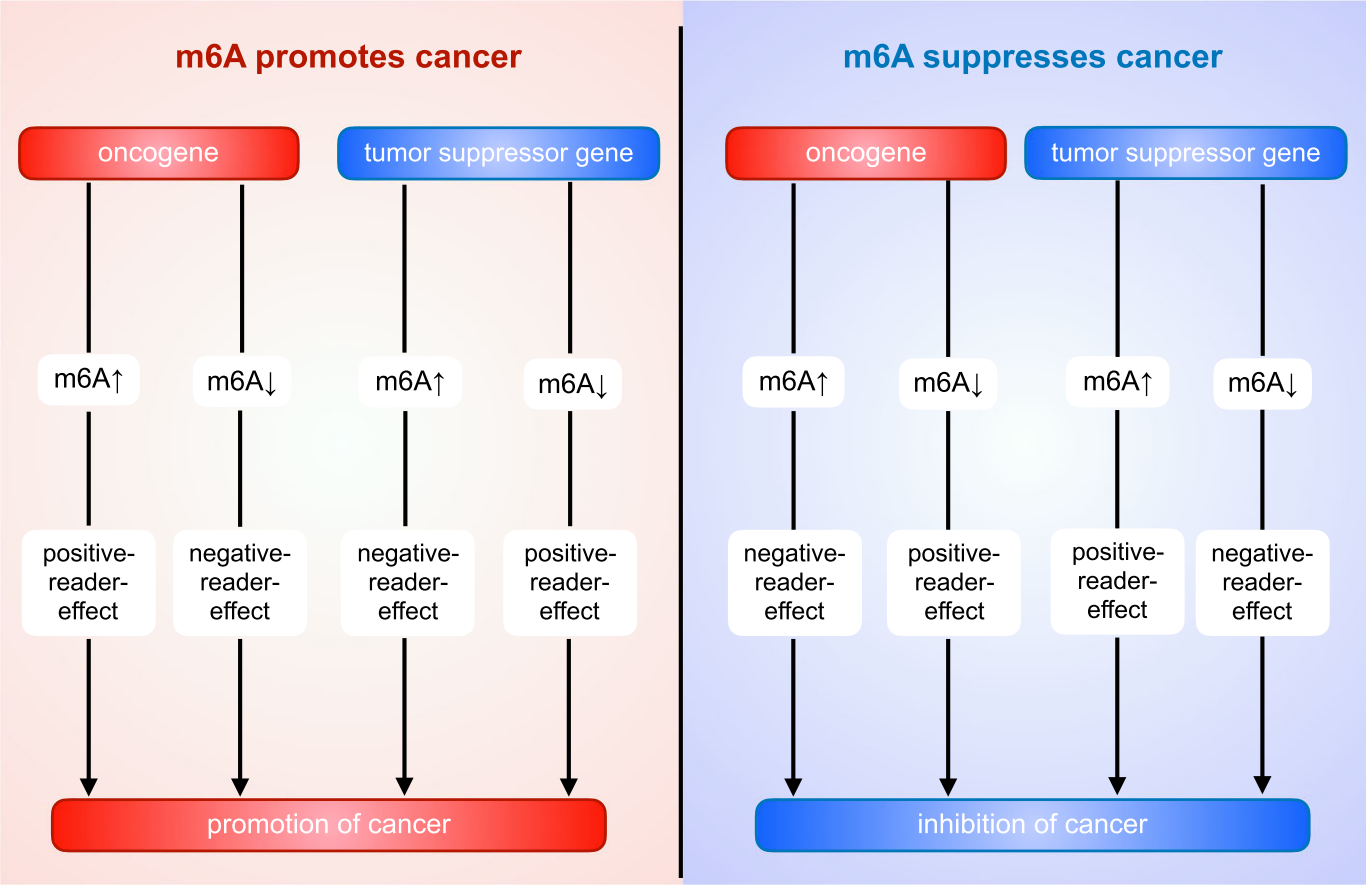
The total effect of m6A on cancer. The total effect of m6A on cancer depends roughly on three elements: (1). Whether the target RNA works as a tumor promoter or a tumor suppressor; (2). The change of m6A level in tumor cells; (3). The post-methylation regulation on target mRNA.

Based on current researches, how m6A influences cancer progression via regulating target genes depends on three elements: (1). Whether the target gene is an oncogene or a tumor suppressor gene (Fig. 4); (2). The aberrant m6A level in cancer (which is decided by the change of expression or activity of writer or eraser); (3). The post-modification regulation on target mRNA (which is decided by readers, both the reader’ s corresponding role to RNA and the change of reader expression and function contribute to this element). The final effect of readers on target mRNA can be divided into two types: positive-reader-role and negative-reader-role. The former is to promote the expression of RNA, while the latter is to inhibit the expression of RNA. More detailed, the positive-reader-role is shown in: 1). Reader promotes target gene translation, splicing, export or stabilization and so on, while the expression of reader is up-regulated or unchanged; 2). Reader promotes the degradation of target mRNA, while the expression of reader is down-regulated. The negative-reader-role is the opposite. For instance, in colorectal carcinoma, the expression of METTL3 is elevated, resulting in higher m6A level in oncogene SOX2. IGF2BP2 is up-regulated and enhances target gene stability, thereby exert a positive-reader-role. Therefore, SOX2 expression is facilitated and m6A plays a cancer-promoting role [80]. In endometrial cancer, the m6A level is lower, leading to inhibited YTHDF1-mediated-tranlation of tumor suppressor gene PHLPP2 and impaired YTHDF2-directed-degradation to oncogene mTORC2, therefore suggesting the cancer-suppressing role of m6A [110].

#### Factors affecting m6A modification and m6A enzymes in cancer

In HCC, miR-145 up-regulates m6A level by targeting 3′ UTR of YTHDF2, leading to suppression of cell proliferation [152].

In non-small-cell lung carcinoma (NSCLC), miR-33a decreases expression of METTL3, thereby attenuating expression of target genes EGFR, TAZ and DNMT3A and suppressing NSCLC cell proliferation [153].

In lung cancer, sumoylation reduces activity of METTL3 catalyzing m6A, resulting in enhanced tumorigenesis [49].

In gastric cancer, up-regulated METTL3 enhances m6A methylation in HDGF and promotes the IGF2BP3-directed-stability of it, therefore facilitating cell proliferation and metastasis in vitro and in vivo. P300-mediated H3K27 acetylation activation in METTL3 promoter promotes transcription of it [111].

In ccRCC, methylenetetrahydrofolate dehydrogenase 2 (MTHFD2) is overexpressed. MTHFD2 enhances m6A modification of HIF-2α and promotes translation of it, thereby resulting in malignant phenotypes [154].

In HCC, high level of nicotinamide N-methyltransferase (NNMT) contributes to vascular invasion and distant metastasis. NNMT inhibits m6A methylation, therefore enhancing CD44v3 formation. NNMT-modulated CD44 m6A methylation improves the RNA stability by impairing ubiquitin-mediated degradation [155].

## Therapeutic strategy based on m6A

Research aimed at uncovering the mechanism has shown that m6A serves as a regulator in cancer [156, 157].

Meclofenamic acid (MA) is one of selective FTO inhibitors by competing with FTO binding sites. As the ethyl ester derivative of MA, MA2 suppresses GSC growth and self-renewal in vitro and inhibits tumor growth in vivo [158, 159].

As another inhibitor of FTO, FB23–2 impairs proliferation and enhances differentiation of AML cells [68].

As a metabolite by mutant IDH1/2 enzymes, R-2-hydroxyglutarate (R-2HG) elevates m6A level and enhances the YTHDF2-mediated-degradation of MYC, ASB2, and RARA [160].

In CRC, carbonic anhydrase IV suppresses Wnt signaling pathway through targeting the WTAP–WT1–TBL1 axis [161].

In epithelial ovarian cancer, elevated m6A level contributes to resistance to Poly (ADP-ribose) polymerase inhibitors (PARPi) through up-regulating Wnt signaling pathway by enhancing stability of FZD10. Restraining Wnt signaling pathway in combination with PARPi therefore may be a potential therapeutic strategy for EOC [162].

In glioblastoma, up-regulated METTL3 promotes GSCs maintenance and glioblastoma progression. Loss of METTL3 results in increased GSCs sensitivity to γ-irradiation [124].

In leukemia, rapidly acquired tolerance to tyrosine kinase inhibitors (TKIs) destroys effective therapy, which proves to be driven by overexpression of FTO. The FTO-mediated demethylation promotes stability of proliferation-related genes [114] Table 3.

**Table 3:** Roles of m6A in RNA metabolism and cancer

tumor types	target RNA	function of target RNA	aberrant expression of m6A enzymes	readers	function of readers	change of target RNA level	reference
AML	MYC, MYB	oncogenic	METTL14↑	EIF3	translation	↑	[58]
Bladder cancer	CDCP1	oncogenic	METTL3↑	YTHDF1	translation	↑	[109]
Colorectal carcinoma	SOX2	oncogenic	METTL3↑, IGF2BP2↑	IGF2BP2	stabilization	↑	[80]
Endometrial cancer	mTORC2	oncogenic	METTL3↓	YTHDF2	degradation	↑	[110]
	PHLPP2	antitumor	METTL3↓	YTHDF1	translation	↓	[110]
Gastric cancer	HDGF	oncogenic	METTL3↑	IGF2BP3	stabilization	↑	[111]
HCC	EGFR	oncogenic	YTHDF2↓	YTHDF2	degradation	↑	[112]
	SOCS2	antitumor	METTL3↑	YTHDF2	degradation	↓	[113]
Leukemia	MERTK,BCL-2	oncogenic	FTO↑	YTHDF2	degradation	↑	[114]
Liver cancer	Snail	oncogenic	METTL3↑	YTHDF1	translation	↑	[115]
Lung cancer	BRD4, EGFR,TAZ	oncogenic	METTL3↑	EIF3	translation	↑	[43, 44]
Ovarian cancer	SRF	oncogenic	IGF2BP1↑	IGF2BP1	stabilization	↑	[78, 100, 101]

HCC: human hepatocellular carcinoma; AML: acute myeloid leukemia

In cervical squamous cell carcinoma (CSCC), FTO enhances the chemo-radiotherapy resistance by up-regulating β-catenin via reducing m6A level [123].

## Conclusion

Epigenetics has become a hot topic in scientific research nowadays [37, 163]. The m6A methylation participates in regulation of cancer malignant phenotype by controlling the expression of cancer-related genes [164–166]. Abnormal level of m6A methylation contributes to tumor pathogenesis and progression. Although m6A has been the focus of many studies in recent years [167, 168], our knowledge about it is far from complete. How expression and activity of readers are regulated in cancer still remains a mistery. The mechanism that readers coordinate their functions since they all bind to m6A but with different functions needs being further elucidated. Little is known about whether there is RNA sequence specificity for readers.

Although m6A enzyme inhibitors have shown tumor-regulating roles in a variety of cancers, more efficacy drugs and novel therapeutic strategies related to m6A are expected to be explored.

## Data Availability

Not applicable.

## Abbreviation

AFF4: AF4/FMR2 family member 4
ALKBH5: Alkb homologue 5
AML: Acute myeloid leukemia
BCSC: Breast cancer stem cell
ccRCC: Clear cell renal cell carcinoma
CEBPZ: CCAAT-box binding factor
CircRNA: Circular RNA
CSCC: Cervical squamous cell carcinoma
ECM: Extracellular matrix
EIF3: Eukaryotic initiation factor 3
EOC: Epithelial ovarian cancer
FTO: Fat mass and obesity-associated protein
GSC: Glioblastoma stem-like cell
HCC: Human hepatocellular carcinoma
HNRNP: Heterogeneous nuclear ribo nucleo protein
HSC: Hematopoietic stem cells
IGF2BPs: Insulin-like growth factor 2 mRNA-binding proteins
LUSC: Lung squamous cell carcinoma
M6A: N6-methyladenosine
METTL3: Methyltransferase-like 3
MTC: Methyltransferase complex
NPC: Nasopharyngeal carcinoma
PARP: Poly (ADP-ribose) polymerase inhibitors
PHLPP2: PH domain leucine-rich repeat protein phosphatase 2
pPA: Premature polyadenylation
PPaRa: Peroxisome proliferator-activator a
RBM15: RNA binding motif 15
SAM: S-adenosylmethionine
SOX2: SRY (sex determining region Y)-box 2
SUMO: Small ubiquitin-like modification
TKI: Tyrosine kinase inhibitors
U6 snRNA: U6 small nuclear RNA
UTR: Untranslated region
WTAP: Wilms Tumor 1 associated protein
XIST: X-inactive specific transcript
YTHDF: YT521-B homology domain-containing protein family
